# High‐throughput single‐cell sequencing of paired TCRα and TCRβ genes for the direct expression‐cloning and functional analysis of murine T‐cell receptors

**DOI:** 10.1002/eji.201848030

**Published:** 2019-05-02

**Authors:** Julia Ludwig, Ann‐Kathrin Huber, Ilka Bartsch, Christian E. Busse, Hedda Wardemann

**Affiliations:** ^1^ Department of B Cell Immunology German Cancer Research Center Heidelberg Germany

**Keywords:** Expression cloning, Functional TCR analysis, Single cell, T cell receptor

## Abstract

Precise clonal and functional assessments of the T cell receptor (TCR) repertoire diversity require paired TCRα and TCRβ gene sequence information at monoclonal level. However, available single‐cell strategies are typically limited in throughput and often do not provide full‐length DNA templates for direct gene cloning. Here, we describe a high‐throughput strategy for the unbiased amplification and automated sequence analysis of paired TCRα and TCRβ genes from primary mouse T cells. The platform links cell phenotype and TCR gene sequence information at single‐cell level. Furthermore, it enables direct functional analyses through the efficient cloning of both genes and the generation of stable TCR expressing cell lines. This highly efficient workflow is a powerful tool to determine the diversity and quality of the murine T‐cell repertoire in various settings, for example in vaccine development, infectious diseases, autoimmunity, or cancer.

## Introduction

αβ T cell receptors (αβ TCRs) are membrane‐bound molecules that recognize peptide antigens presented on major histocompatibility complexes (MHCs). The TCRα and TCRβ chains are encoded by genes that are assembled from a large number of small gene segments during early T‐cell development in the thymus. In contrast, the number of TCR gene segments used by γδ T cells, a specialized T cell subset with more innate‐like features, is much lower and their usage is developmentally controlled 1, 2. The random and imprecise nature of the somatic recombination process and the high combinatorial diversity preclude reliable predictions of the peptide specificity for any given αβ TCR. High‐throughput sequencing of rearranged TCR genes has widely been used to assess the diversity of αβ T cell repertoires under various conditions 3, 4, 5, 6. The ultimate goal is to be able to predict antigen‐specificity and TCR function based on sequence analysis. Bulk sequencing approaches to capture the diversity of αβ T cell repertoires typically focus on TCRβ complementarity determining region 3 (CDR3) analyses. However, precise clonal and functional assessments require paired TCRα and TCRβ gene information 7, 8, 9. Single‐cell repertoire analyses can overcome these limitations. Recent technical advances have enabled high‐throughput measurements at single‐cell level, but typically do not provide information on the cell phenotype 10. Further, full‐length gene products for direct expression cloning are typically not readily available to enable functional TCR analyses. Here, we describe a platform for the high‐throughput analysis of murine αβ‐TCR repertoires at single‐cell level that integrates cell phenotype with TCR gene sequence information and enables functional TCR assessments by direct expression cloning of paired TCRα and TCRβ genes and the generation of stable TCR transgenic cell lines.

## Results

### High‐throughput paired αβ TCR transcript amplification and sequencing from defined T‐cell subsets

**Figure 1:**
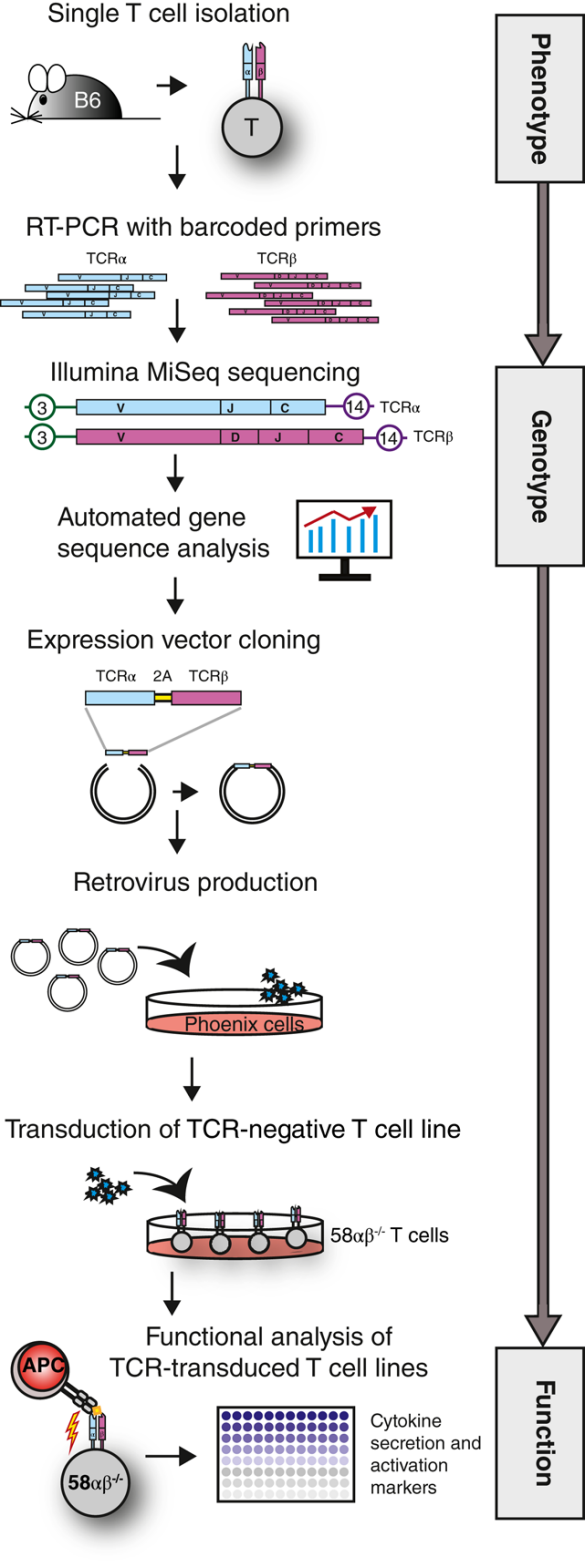
Schematic overview of the murine single‐cell TCR analysis platform. T cell subpopulations are identified by flow cytometry and single T cells are isolated using the indexed single‐cell sort option to record the phenotypic parameters of each cell. Total RNA is reverse transcribed. cDNA is used to amplify TCRα and TCRβ transcripts with barcoded primers (exemplarily shown as tag number “3” and “14”). All PCR amplicons were pooled and sequenced using NGS (Illumina MiSeq). TCR V gene usage is assessed using an automated analysis pipeline (sciReptor). TCR genes are cloned into a retroviral expression vector. Retroviral particles are produced in Phoenix Eco cells. Over‐night cell culture supernatants are collected and used for the transduction of CD4‐ or CD8‐positive 58αβ^−/−^ T cells lacking the expression of an endogenous TCR. Starting 1–2 days after the transduction, 58αβ^−/−^ T cells show TCR surface expression and are stimulated with antigen‐loaded APCs. Different parameters such as IL‐2 secretion or surface activation marker expression serve as functional read‐outs.

**Figure 2:**
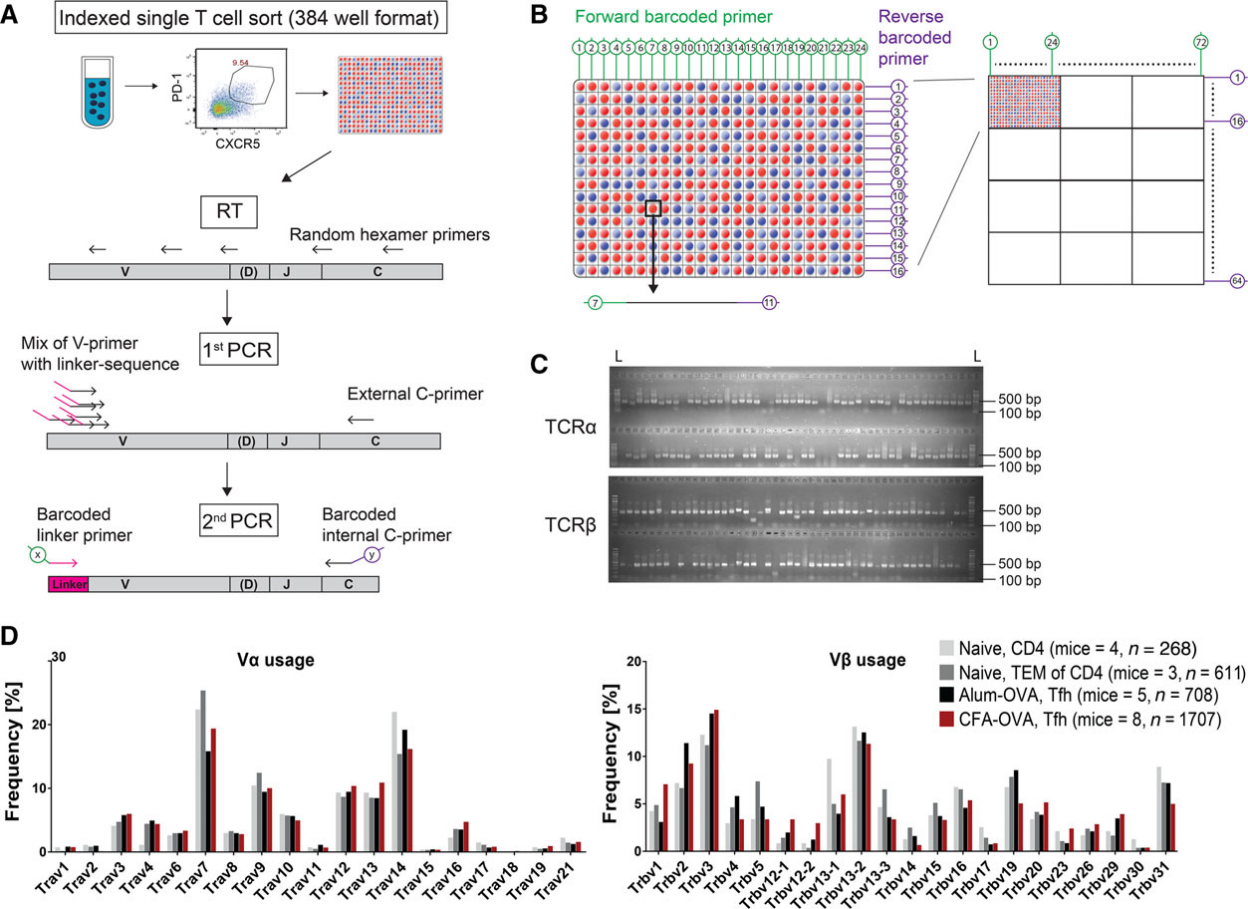
Single‐cell based murine TCRα and TCRβ gene transcript amplification. (A) Schematic view of the T cell isolation and TCR gene amplification strategy. Single T cells from defined subpopulations are isolated into 384‐well plates using indexed flow cytometric cell sorting. RNA is reverse transcribed using random hexamer primers. TCRα and TCRβ genes are amplified from cDNA in two independent semi‐nested PCR reactions. In the first PCR, a cocktail of V gene specific forward primers in combination with a reverse C region primer is used. All forward primers carry a 5′ linker sequence to generate a universal primer‐binding site. In the second PCR, barcoded versions of the linker primer and of a nested C region primer are used. (B) Schematic presentation of the primer matrix setup. Barcoded forward (column) and reverse (row) primers generate amplicons with unique barcode combinations for each well position to allow for the parallel sequencing of pooled amplicons from 12 384‐well plates. (C) TCRα (∼450 bp) and TCRβ (∼480 bp) gene amplification efficiency as determined by gel electrophoresis. L indicates the molecular weight marker. Gel picture represents one out of at least five independent experiments. (D) TCRα and TCRβ V gene usage analysis of sciReptor‐processed sequences from the indicated T cell subpopulations of naïve or OVA‐immunized mice. Sequences from three independent experiments were pooled and the number of mice and analyzed sequences (*n*) is indicated for each group.

To assess the expressed TCR repertoire of phenotypically defined mouse T‐cell populations at single‐cell level, we designed a strategy for the high‐throughput amplification and sequencing of paired TCRα and TCRβ genes. The strategy enables direct TCR expression cloning and allows the integration of phenotypic, gene sequence, and functional datasets at monoclonal level (Fig. 1). Individual T cells from five different subpopulations were isolated into 384‐well plates by flow‐cytometric single‐cell sorting. Concomitantly, the phenotypic parameters of each individual cell were recorded using the indexed cell‐sorting option (Fig. 2). cDNA was prepared using random hexamer primers. Subsequently, TCRα and TCRβ transcripts were amplified in two independent semi‐nested PCRs. To amplify complete or nearly complete V genes, minimal sets of forward primers with annealing sites in the 5′‐end of framework region 1 (FWR1) were designed (Supporting Information Tables S1 and S2). Cocktails of these V gene‐specific forward primers in combination with the respective TCRα or TCRβ constant (C) region reverse primers were used in the first PCR (Fig. 2A). All individual V gene forward primers contained a common linker sequence 7. For amplification in the second PCR, a forward primer with sequence homology to the universal linker and a nested TCRα or TCRβ C region reverse primer were used. All second PCR primers contained barcodes to allow for the pooling of all amplicons and next generation sequencing (NGS; Fig. 2B). To minimize the number of uniquely barcoded primers and thereby costs, we designed a two‐dimensional primer matrix that tagged each PCR product with a row‐ and column‐specific barcode 11. Gel electrophoresis confirmed the efficient amplification (Fig. 2C). To obtain TCRα and TCRβ gene information, all second PCR products were pooled and sequenced using Illumina MiSeq. The sequence data were then processed using an updated version of sciReptor (Supporting Information Fig. S1), a sequence analysis pipeline that we previously developed for the analysis of Ig repertoires at single‐cell level 12. Assessments of the TCRα and TCRβ V gene usage showed that all functional TCRα and TCRβ V gene families had been amplified with our primer sets (Fig. 2D). In total, we amplified 79 of 121 and 26 of 26 known functional TCRα and TCRβ gene segments, respectively. The relative frequency of the individual V genes was similar in all T‐cell subpopulations. Paired TCRα and TCRβ sequence information was obtained from 45 to 65% of input cells with no strong difference between activated or non activated cell populations or among cells from different tissues (Table 1). Thus, our strategy enabled the unbiased and efficient amplification and analysis of paired TCRα and TCRβ transcripts from different mouse T‐cell subpopulations and tissues.

**Table 1:** Combined TCRα and TCRβ gene amplification and sciReptor annotation efficiency

Cell of origin			Efficiency (%)		
Population, tissue/organ	Phenotype	Number of experiments, number of cells	TCRα	TCRβ	TCRαβ
CD4, spleen	CD4^+^	3, *n* = 1536	55–65	65–75	45–55
Activated CD4, spleen	CD4^+^CD44^+^CD62L^low^	3, *n* = 1152	58–67	62–76	45–60
T follicular helper, LNs	CD4^+^CD44^+^CD62L^low^ CXCR5^+^PD‐1^+^	3, *n* = 3456	58–67	62–76	45–60
T regulatory, LNs	CD4^+,^ GFP^+^ (FoxP3^+^)	2, *n* = 2304	60–70	80–85	55–60
Activated CD8, liver	CD3^+^CD8^+^PD‐1^+^	2, *n* = 2688	73–75	65–67	49–52

### Cloning and expression of paired TCRα and TCRβ genes from single T cells

**Figure 3:**
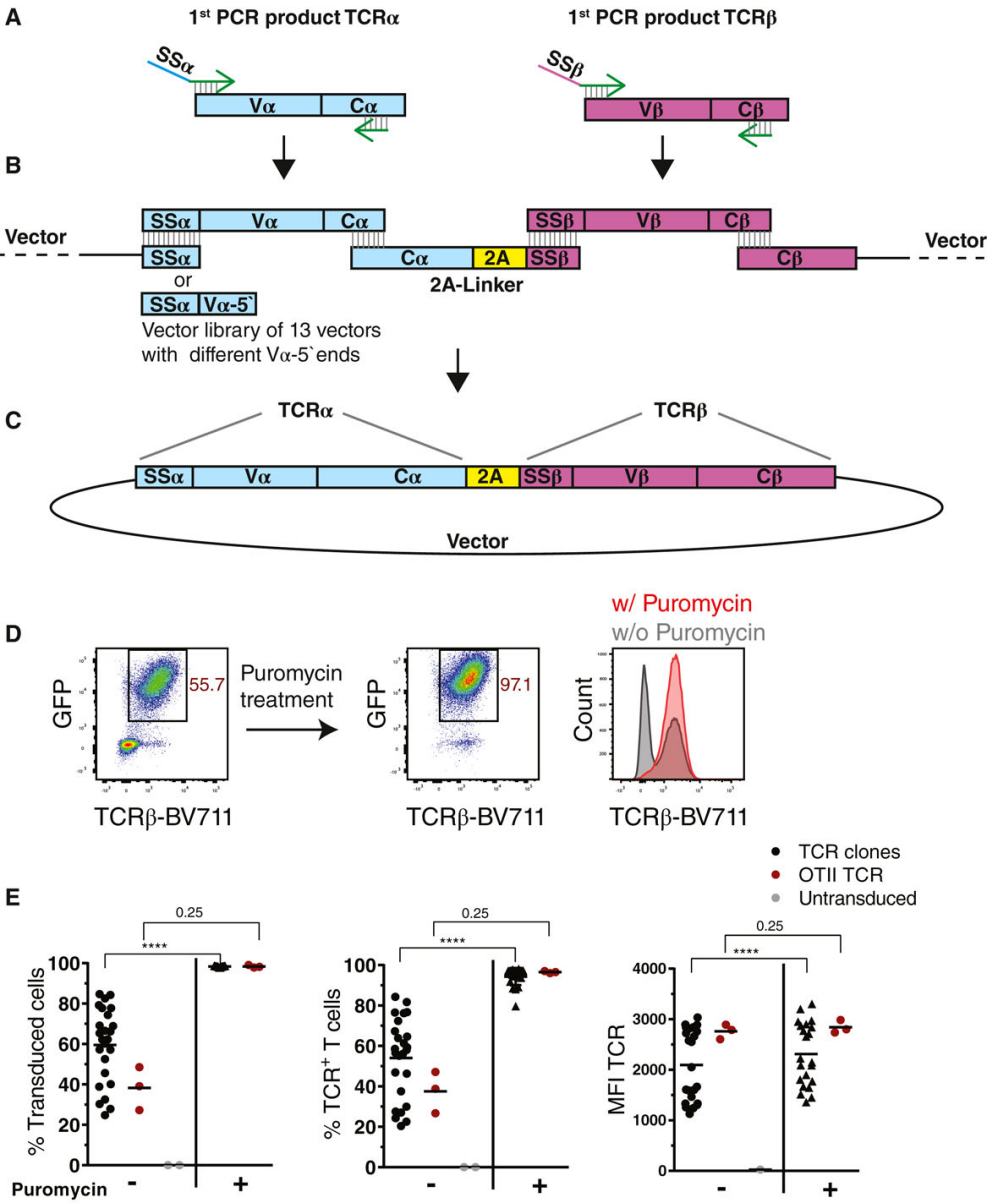
Cloning and expression of TCR genes. (A) Schematic presentation of the PCR strategy to generate TCRα and TCRβ gene fragments with 5′ sequence homology to signal peptides of TCRα (SSα) or TCRβ (SSβ) using V‐gene‐specific and nested C‐region primers and the first PCR product as template (Supporting Information Table S3 and S4). (B) Schematic presentation of the Gibson assembly strategy to clone the TCRα and TCRβ gene fragments (A) separated by the 2A‐linker construct head‐to‐tail into the respective linearized retroviral expression vector from a library of vectors encoding the 5′‐end of different Vα gene segments (Supporting Information Table S5). Sequence homology regions that allow the assembly in a single reaction are indicated for each DNA fragment. (C) Schematic view of the final expression vector containing the complete TCRα and TCRβ genes with signal peptides separated by the 2A peptide. (D) Representative flow cytometric gating strategy for the analysis of TCR surface expression levels before and after puromycin treatment of retrovirally transduced live T cells based on GFP expression and TCRβ staining (full gating strategy shown in Supporting Information Fig. S3). (E) Transduction efficiency (GFP^+^), frequency of TCR expressing cells (GFP^+^TCRβ^+^), and TCR expression levels (TCRβ MFI of GFP^+^) based on flow cytometric analyses before and after puromycin treatment for T‐cell lines expressing TCRs cloned from primary mouse CD4 T cells after OVA immunization in comparison to the OTII TCR as shown in (D) 15. Pooled data from two independent experiments are shown. Indicated groups were compared using Wilcoxon matched‐pairs signed rank test (****p < 0.0001, or *p*‐value directly indicated in graph).

To facilitate functional TCR assessments, we cloned the paired TCRα and TCRβ amplicons and expressed them in a TCR‐negative hybridoma T‐cell line (Fig. 3). To promote the equimolar expression of both TCR chains, the TCRα and TCRβ genes were inserted in head‐to‐tail configuration into a single retroviral expression vector and connected via a porcine teschovirus‐1 2A peptide (2A) linker 13. Gibson assembly was used to combine the TCRα and TCRβ amplicons with the 2A linker fragment and the linearized vector based on sequence homology (Supporting Information Fig. S2). First, TCR genes with overlapping regions to the vector were generated by PCR using V‐gene and C‐region specific primers and the first PCR product as template (Fig. 3A; Supporting Information Tables S3 and S4). This PCR also served to revert potential errors introduced by the degenerated primers in the first PCR. Second, the linker fragment containing the TCRα constant region (Cα) and TCRβ signal sequence (SSβ) linked via the 2A peptide sequence was generated (Fig. 3B), as well as a library of 13 vectors containing the TCRα signal sequence (SSα) and TCRβ constant region (Cβ) (Supporting Information Table S5). All fragments were combined in a single reaction (Fig. 3C; Supporting Information Fig. S2) and sequenced to confirm their sequence identity and in‐frame assembly. Viral particles were produced in Phoenix Eco cells and the supernatants were used to transduce TCR‐negative 58αβ^−/−^ T cells stably expressing CD4 or CD8, depending on the cell of origin from which the TCR was cloned 14. The successful transduction was monitored by GFP expression independently of the TCR expression (Fig. 3D and E; Supporting Information Fig. S3). Puromycin treatment lead to the strong enrichment of transduced TCR‐positive cells (Fig. 3E). TCR expression levels varied between different TCRs and increased only slightly (on average 10%) by puromycin treatment.

In summary, we developed a highly efficient system to clone and express murine TCR genes and to generate stable TCR‐positive CD4 and CD8 cell lines from single primary T cells.

### TCR reactivity

**Figure 4:**
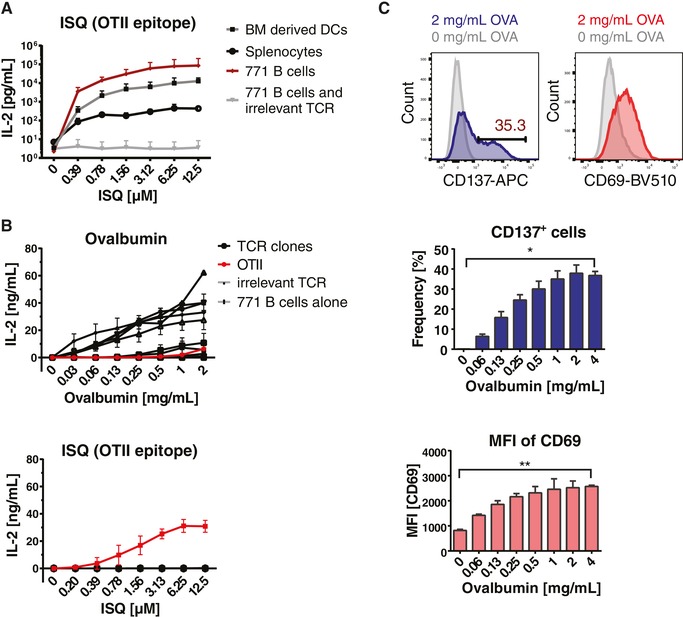
TCR reactivity analysis. (A) IL‐2 secretion of transduced CD4^+^ 58αβ^−/−^ T cells expressing OTII or a non‐OVA reactive control TCR, cloned from a naïve mouse, 24 h after coculture with ISQ peptide pulsed BM‐derived DCs, total splenocytes, or 771 B cells as antigen presenting cells. Data show means and upper SD from five independent experiments for BM‐derived DCs and two independent experiments for the other groups. (B) IL‐2 secretion of transduced CD4^+^ 58αβ^−/‐^ T cells expressing 30 different TCRs cloned from primary T cells of five different OVA‐immunized mice after stimulation with OVA (top) or ISQ peptide (bottom) pulsed 771 B cells. The culture of 771 B cells alone or with a non‐OVA‐reactive control TCR served as negative controls. Data show means + SD from two independent experiments with two technical replicates each. (C) CD137 and CD69 surface expression levels after coculture of CD4^+^ 58αβ^−/−^ T cells expressing a representative OVA‐reactive TCR cloned from primary mouse T cells after OVA immunization with OVA‐pulsed or nonpulsed 771 B cells. The frequency of CD137^+^ cells and MFI for CD137 of transduced CD4^+^ 58αβ^−/−^ T cells is indicated. Data show means + SD from at least two independent experiments with two technical replicates each. Indicated data were compared using paired *t*‐test (*p < 0.05, **p < 0.005).

To determine whether the T cell lines allowed functional assessments of the TCR quality, we compared 30 different lines generated from CD4^+^CXCR5^+^ T follicular helper cell (Tfh) cells of C57BL/6 mice immunized with OVA based on their IL‐2 secretion after antigen‐mediated stimulation. For comparison, we also cloned and expressed the well‐characterized OTII TCR with specificity for the ISQ OVA peptide 15. OTII TCR‐transduced cells secreted IL‐2 upon coculture with ISQ‐pulsed total splenocytes as well as bone marrow (BM) derived dendritic cells (DCs) as antigen presenting cell (APC) (Fig. 4A). High IL‐2 levels were also produced with 771 cells as APC, a B6‐derived B cell line (771), which is characterized by strong MHCII expression 16. Due to their easy handling compared to primary cells, we used 771 cells to compare the OVA response of transgenic OTII TCR cells to the response of cell lines expressing TCRs cloned from primary Tfh cells. Nearly half of the Tfh TCRs (12/30; 40%) responded to OVA stimulation by the secretion of IL‐2 in a dose‐dependent manner (Fig. 4B). The responsiveness was different in lines expressing different TCRs, indicating differences in their affinity and or specificity. The level of IL‐2 secretion ranged from 0.5–80 ng/mL in response to 2 mg/mL OVA and from 0.05–30 ng/mL in response to 0.25 mg/mL OVA, demonstrating the broad range of responsiveness. Of note, only the OTII TCR, but none of the Tfh TCRs responded to the ISQ peptide.

Not only IL‐2 secretion was upregulated in an antigen‐mediated dose‐dependent manner, but also expression of the activation markers CD69 and CD137 (Fig. 4C). Individual TCRs behaved similarly in IL‐2 secretion and upregulation of CD69 and CD137 (Supporting Information Fig. S4).

We conclude that our transduced cell lines express functional TCRs and can be used for functional and qualitative assessments of the receptors.

## Discussion

In this study, we have established a platform for the unbiased characterization of the murine TCR repertoire at phenotypic, gene sequence, and functional level. The platform described here is ideally suited for the deep sampling of small phenotypically and anatomically defined T‐cell populations, where all available cells can be sorted and analyzed (e.g. follicular Tfh cells from lymph nodes). The physical separation of the cells by flow cytometry enables sequence linkage to the cell phenotype and at the same time allows efficient TCR chain pairing.

Commercial platforms that provide physical linkage of transcripts in a microfluidic context, e.g. DropSeq 17, have only recently been developed for the analysis of mouse TCRs. The encapsulation process is based on limiting dilution and follows a Poisson distribution. Therefore, the reliable encapsulation of only one cell per droplet requires low λ. DNA‐barcoded antibodies have recently been developed to link cell phenotype information with droplet‐based amplification and sequencing methods and show great future potential. However, at the moment, flow cytometric cell sorting approaches still provide higher resolutions 18.

A great advantage of our platform is that it allows direct TCR gene expression cloning compared to droplet‐based sequencing methods, which require expensive gene syntheses for functional TCR assessments. The use of Gibson assembly rather than conventional cloning protocols and the highly efficient puromycin‐mediated selection of TCR‐positive cells enabled the fast completion of the workflow from cell sorting to functional assays. Furthermore, the small 384‐well reaction volume, the compatibility with NGS, and the possibility of direct parallel cloning of individual TCRs in 96‐well format make our approach highly cost‐efficient and advanced over existing protocols 8, 10, 19, 20.

In summary, the platform facilitates the rapid functional characterization of murine αβ TCRs from primary T cells. It provides a powerful tool to assess αβ TCR repertoire diversity at qualitative level in a highly efficient cell‐line‐based APC coculture system for a variety of different settings, for example vaccination, cancer, autoimmunity, or infectious diseases.

## Materials and methods

### Mouse housing and immunization of mice

C57BL/6J mice (Jackson laboratories) were bred at the central animal facility of the German Cancer Research Center (Heidelberg, Germany). All animals were kept under specific pathogen‐free conditions. Experiments were approved by the regional council Karlsruhe and conducted according to governmental and institutional guidelines. C57BL/6J mice were immunized s.c. with 100 µg Ovalbumin (OVA) (Invivogen) adsorbed to Alum or emulsified in CFA. Each mouse received 50 µL suspension at the right and left side of the base of tail. Ten days post immunization, single‐cell suspensions from spleens and lymph nodes were prepared by standard procedures and analyzed directly or cryopreserved for later analyses.

### Isolation of mouse T cells by flow cytometric single‐cell sorting

Single‐cell suspensions from spleens and lymph nodes of OVA‐immunized mice were incubated for 15 min at 4°C with anti‐CD16/CD32 Fc‐receptor block (2.4G2, eBiosciences), followed by incubation for 45 min at 4°C with anti‐CXCR5‐PE (L138D7, Biolegend), anti‐CD62L‐PE‐Cy7 (MEL‐14, Biolegend), anti‐PD‐1‐BV605 (J43, BD Biosciences), anti‐CD4‐FITC (GK1.5, Biolegend), anti‐CD44‐BV785 (IM7, Biolegend), and anti‐ICOS‐BV421 (7E.17G9, BD Biosciences) antibody, all diluted in PBS containing 2% FCS. Dead cells were excluded using 7‐Aminoactinomycin D (7AAD, life technologies). Single T cells from defined subpopulations were isolated into 384‐well plates containing 2 µL/well of ice‐cold Sort/RHP mix (4 mM DTT, 0.69% NP‐40, 20 ng/µL random‐hexamer‐primers (RHP, Roche), 1.87 U/µL RNAsin (Promega) in 0.25× PBS) with an AriaIII cell sorter and index‐sort option (BD Biosciences).

### cDNA synthesis and TCR gene transcript amplification

Subsequently 384‐well plates containing sorted cells in Sort/RHP mix were incubated at 68°C for 1 min and cooled on ice before addition of 2 µL/well RT‐mix containing 2× first strand buffer (Invitrogen), 15 mM DTT (Invitrogen), 1.6 mM dNTPs each (Invitrogen), 1.1 U/µL RNAsin (Promega), and 6.9 U/µL SuperScript IV (Invitrogen). cDNA was synthesized (42°C for 5 min, 25°C for 10 min, 50°C for 60 min, and 94°C for 5 min) and used for the independent amplification of TCRα and TCRβ genes in two semi‐nested PCRs. TCRα and TCRβ first and second PCR primers are listed in Supporting Information Table S2 and S3, respectively. All PCR reactions were performed in 384‐well plates in a total volume of 10 µL per well. Primary PCRs were performed with 0.625 µL of template cDNA, 1× HotStar Taq buffer (Qiagen), 75 nM of each Vα or Vβ forward primer, 100 nM of Cα or Cβ reverse primer, 0.2 mM of each dNTP (Invitrogen), and 0.03 U/µL HotStar Taq (Qiagen) at 94°C for 10 min followed by 50 cycles of 94°C for 40 s, 62°C for 45 s, 72°C for 1 min, and a final incubation at 72°C for 10 min. Secondary PCRs were performed with 1 µL of unpurified primary PCR product as template, 1× HotStar Taq buffer (Qiagen), 167 nM of forward and reverse primers containing Matrix‐PCR barcodes (as described before 11, 21), 0.2 mM of each dNTP (Invitrogen), 1 mM additional MgCl_2_ (New England Biolabs), and 0.03 U/µL HotStar Taq (Qiagen) at 94°C for 10 min followed by 50 cycles of 94°C for 30 s, 56°C for 30 s, 72°C for 55 s, and a final incubation of 72°C for 10 min. PCR products were analyzed on 2% agarose gels and pooled for Illumina MiSeq (2 × 300 bp) sequencing.

### TCR repertoire analysis

Raw paired‐end reads were assembled using PandaSeq 2.8 22, selecting reads of a quality higher than 0.8 and an assembled sequence length of 480–560 bp. Around 50 000–100 000 reads per 384‐well plate were randomly selected for further processing. Data analysis was performed using sciReptor 12 further enhanced for mouse TCR analysis (version tag “v1.1‐0‐g6ac6c8d,” freely available at https://github.com/b-cell-immunology/sciReptor).

### Gene‐specific TCR amplification

TCR V gene‐specific PCRs were performed to generate overlapping regions with the vector and 2A linker (Fig. 3) that allow direct expression vector cloning by Gibson assembly. V‐gene‐specific PCRs were performed at 94°C for 30 s followed by 35 cycles of 94°C for 10 s, 64°C for 30 s, 72°C for 30 s, and a final incubation at 72°C for 10 min in 96‐well plates and a total volume of 20 µL per well. All reactions contained 0.5 µL primary PCR product as template, 0.5 µM forward and reverse primer (Supporting Information Table S4 and S5), 0.25 mM of each dNTP (Invitrogen), 1× Phusion PCR buffer, and 1 U/µL Phusion polymerase (New England Biolabs). PCR products were purified using the 96‐well NucleoSpin Gel and PCR Clean‐Up Kit (Macherey–Nagel).

### TCR cloning

The 2A‐linker sequence was excised from the pEX‐K4 vector by restriction digest with EcoRI and XbaI. All expression vectors were linearized in a single‐cut restriction reaction using BamHI‐HF. Vector and 2A‐linker DNA were separated on agarose gels, excised, and purified using the NucleoSpin Gel and PCR Clean‐Up Kit (Macherey–Nagel).

NEBuilder HiFi DNA Assembly Master Mix (New England Biolabs) was used to assemble 0.025–0.05 pmol of the 2A‐linker and the TCRα and TCRβ amplicons (from the gene‐specific PCR described above) with 0.025 pmol of vector DNA in a total reaction volume of 10 µl for 60 min at 50°C. Chemically competent *Escherichia coli* DH10b (Invitrogen) were transformed with 3 µL of the reaction product via heat shock at 42°C for 45 s and plated onto 100 µg/mL ampicillin containing LB Broth with agar (Sigma) plates. Individual ampicillin‐resistant bacterial colonies were picked and dipped into a PCR mastermix containing 1× Hot Star Taq PCR buffer, 0.25 mM dNTPS each, 0.5 µM forward and reverse primer (TCR_control_fw 5′‐TCGATCCTCCCTTTATCCAG‐3′, TCR_control_rev 5′‐CCATGGAACTGCACTTG‐3′), and 0.5 U/µL HotStar Taq (Qiagen). PCRs were performed at 94°C for 10 min followed by 35 cycles of 94°C for 30 s, 55°C for 30 s, 72°C for 2 min, and a final incubation of 72°C for 10 min. PCR products of the expected size (1600–1700 bp) were Sanger sequenced to confirm their identity with the secondary PCR products and to exclude PCR and cloning errors. Positively screened bacterial clones were grown in 5 mL LB medium (Sigma) containing 75 µg/mL Ampicillin for 16 h at 37°C under continuous shaking (220 rpm). Plasmid DNA was purified using a plasmid purification kit (Macherey–Nagel) in 96‐well format.

### Virus production and retroviral transduction of 58αβ^−/−^ T cells

Phoenix Eco cells (Allele Biotech) were cultured in six‐well plates (TPP) under standard conditions of 10% heat‐inactivated FCS (Life Technologies) in DMEM (Gibco) and grown to 60–80% confluence at 37°C and 8% CO_2_. Transfections were performed using CaCl_2_. A total of 10 µL DNA (0.2 µg/µL) were incubated at 55°C for 30 min before adding 12.5 µL CaCl_2_ (2.5 M) and 102.5 µL H_2_0. Briefly, 125 µL 2× HBS (pH 7.05) were added dropwise while vortexing and the solution was kept at RT for 10 min. The medium of confluent Phoenix Eco cells was changed to 2 mL fresh medium containing 25 µM Chloroquine (Sigma) per well. The DNA/CaCl_2_ solution (250 µL/well) was added dropwise and incubated for 6 h. Afterward, the medium was changed to 58αβ^−/‐^ medium consisting of DMEM, 10% FCS, 1 mM sodium pyruvate (Life Technologies), and 0.05 mM β‐mercaptoethanol (Life Technologies). After 16 h, supernatants containing viral particles were collected. Cell debris were removed by spinning at 1000 × *g* for 5 min. CD4^+^ 58αβ^−/−^ cells (2.5 × 10^4^) were resuspended in 200 µL of the supernatants and 10 µg/mL protamine sulfate (MP Biomedicals) and plated into flat bottom 96‐well plates (TPP; 2.5 × 10^4^/well). Spin transfection was performed at 2000 × *g* and 32°C for 2 h. A total of 100 µL/well of fresh medium was added and cells were incubated for 48 h. Cells were incubated for 2–3 days with medium containing 0.8 µg/mL puromycin dihydrochloride (Sigma) to select for transduced cells.

### Stimulation of TCR transduced 58αβ^−/−^ T cells

BM‐derived dendritic cells from C57BL/6 mice (0.75 × 10^5^/well), splenocytes from C57BL/6 mice (4 × 10^5^/well), and 771 B cells (0.75 × 10^5^/well) B cells 16 were loaded with the indicated concentrations of OVA (Invivogen) or ISQ peptide (Invivogen) in 150 µL/well 58αβ^−/−^ medium (96‐well plate, U‐bottom, TPP) at 37°C and 8% CO_2_ overnight. The next day, 0.5 × 10^5^ transduced 58αβ^−/−^ T cells in 50 µL medium were added per well. After 24 h of incubation, supernatants were harvested for IL‐2 immunoassay and 58αβ^−/−^ cells for FACS analyses.

### IL‐2 immunoassay

Nunc F96 Micro Well PolySorp Black (Thermo Fisher) microplates were coated over night at 4°C with 1 µg/mL rat anti‐mouse IL2 capture antibody (Biolegend, Clone JES6‐1A12) in sodium carbonate buffer (50 mM Na_2_CO_3_, 50 mM NaHCO_3_, pH 9.6), washed twice with PBS‐T (0.05% Tween20 in PBS), and blocked with 0.2% gelatin in PBS. Plates were washed five times with PBS‐T. Culture supernatants were added and incubated overnight at 4°C. Plates were washed five times and incubated with 0.25 µg/mL anti‐mouse IL2‐biotin detector antibody (Biolegend, Clone JES6‐5H4) for 2 h at room temperature. After five more wash steps with PBS‐T, 0.1 µg/mL DELFIA europium (Eu)‐labeled streptavidin (PerkinElmer) diluted in IL‐2 assay buffer (PerkinElmer) was added and incubated for 45 min at room temperature. The reaction was enhanced with DELFIA enhancement solution (PerkinElmer). After 10 min of incubation at room temperature, fluorescence values were measured by using 320 nm excitation and 615 nm emission fluorescence filters in a spectrofluorometer (Tecan, Infinite M1000 Pro). IL‐2 secretion curves were analyzed and AUC values calculated by GraphPad Prism Version 6. TCR clones with AUC values above 0.5 (50× increased in comparison to negative controls; 0.01 ± 0.003) were considered OVA reactive.

### Flow cytometric analysis

TCR surface expression levels and activation marker expression of transduced 58αβ^−/−^ T cells were assessed by flow cytometry after incubating with anti‐TCRβ‐chain‐BV711 (clone H57‐597, Biolegend), anti‐CD69‐BV510 (clone H1‐2F3, Biolegend), and anti‐CD137‐APC (clone 17B5, Biolegend) diluted in PBS containing 2% FCS for 45 min at 4°C. 7AAD (Life technologies) was used to exclude dead cells. Flow cytometric analysis was performed according to the guidelines for the use of flow cytometry and cell sorting in immunological studies 23. Data acquisition was carried out with an AriaIII (BD Biosciences) and analyzed with FlowJo Version 10.4.1 (Tree Star).

## Conflict of interest

The authors declare no financial or commercial conflict of interest.

**Abbreviations**
AUC: area under curve
FWR: framework region
NGS: next‐generation sequencing
SS: signal sequence
Tfh: T follicular helper cell

## Supporting information

Supporting Information
