# Supplementary material for: High‐throughput single‐cell sequencing of paired TCRα and TCRβ genes for the direct expression‐cloning and functional analysis of murine T‐cell receptors
Source: Eur J Immunol. 2019 May 2;49(8):1269–77. doi: 10.1002/eji.201848030 (PMC6767390; doi:10.1002/eji.201848030)
Supplement: Supplementary file 1 — Supporting Information [file EJI-49-1269-s001.pdf]

European Journal of Immunology

Supporting Information
for
DOI 10.1002/eji.201848030

Julia Ludwig, Ann-Kathrin Huber, Ilka Bartsch, Christian E. Busse and Hedda Wardemann

High-throughput single-cell sequencing of paired TCRα and TCRβ genes for the direct expression-cloning and functional analysis of murine T-cell receptors

© 2019 The Authors. *European Journal of Immunology* published by WILEY-VCH Verlag GmbH & Co. KGaA, Weinheim.

Supporting information

Supporting Table S1: TCRα 1st and 2nd PCR primers

PCR	Name	5´-3´ nucleotide sequence
1st PCR	TRAC-REVex	CGGCACATTGATTTGGGAGTC
2nd PCR	TRAC-REVin	CTGTCCTGAGACCGAGGATC
1st PCR	Trav1	CCAGGGTTTTCCCAGTCACGACCAGGGTGTGGAGCAGCCTGCCAA
	Trav2/21	CCAGGGTTTTCCCAGTCACGACATCTATTGGTACCGACAGGTTCC
	Trav3	CCAGGGTTTTCCCAGTCACGACGGCGAGCAGGTGGAGCAGCGC
	Trav4	CCAGGGTTTTCCCAGTCACGACTCTGCTCTGAGATGCAATTTT
	Trav5	CCAGGGTTTTCCCAGTCACGACCTACTTCCCTTGGTATAAGCAAGA
	Trav6	CCAGGGTTTTCCCAGTCACGACACCCAACTCTKTTCTGGTATGT
	Trav7	CCAGGGTTTTCCCAGTCACGACAAGGTACAGCAGAGCCCAGAATC
	Trav8	CCAGGGTTTTCCCAGTCACGACCCTGAGCATCCACGAGGGTGAA
	Trav9	CCAGGGTTTTCCCAGTCACGACAGCTGAGATGCAASTATTCCT
	Trav10	CCAGGGTTTTCCCAGTCACGACCATGGAGAGAAGGTCGAGCAACA
	Trav11	CCAGGGTTTTCCCAGTCACGACAAGACCCAAGTGGAGCAGAGTC
	Trav12	CCAGGGTTTTCCCAGTCACGACGTGACCCAGACAGAAGGCCTGG
	Trav13	CCAGGGTTTTCCCAGTCACGACGTCCTTGGTTCTGCAGGAGG
	Trav14	CCAGGGTTTTCCCAGTCACGACCAGCAGCAGGTGAGACAAAG
	Trav15	CCAGGGTTTTCCCAGTCACGACCTGGACTGTTCATATGAGACAAGT
	Trav16	CCAGGGTTTTCCCAGTCACGACAGAAGGTAACACAGACTCAGAC
	Trav17	CCAGGGTTTTCCCAGTCACGACCAGTCCGTGGACCAGCCTGATGC
	Trav18	CCAGGGTTTTCCCAGTCACGACGAGCAGAGTCCTCGGTTTCTGAG
	Trav19	CCAGGGTTTTCCCAGTCACGACCCAGCAAGTTAAACAAAGCTCTCC
	Trav23	CCAGGGTTTTCCCAGTCACGACCCTCCGTTTCTCGGCTCCTGG
2nd PCR	Linker	CCAGGGTTTTCCCAGTCACGAC

Purple letters indicate the linker sequence [7].

Supporting Table S2: TCRβ 1st and 2nd PCR primers

PCR	Name	5´-3´ nucleotide sequence
1st PCR	TRBC-REVex	GGTAGCCTTTTGTTTGTTTGC
2nd PCR	TRBC-REVin	TGATGGCTCAAACAAGGAGACC
1st PCR	Trbv01	CCAGGGTTTTCCCAGTCACGACGTGACTTTGCTGGAGCAAAACCC
	Trbv02	CCAGGGTTTTCCCAGTCACGACGACCCGAAAATTATCCAGAAACC
	Trbv03	CCAGGGTTTTCCCAGTCACGACGGACCCAAAGTCTTACAGATCCC
	Trbv04	CCAGGGTTTTCCCAGTCACGACGAGACGGCTGTTTTCCAGACTCC
	Trbv05	CCAGGGTTTTCCCAGTCACGACAACACTAAAATTACTCAGTCACC
	Trbv12	CCAGGGTTTTCCCAGTCACGAC GATTCTGGGGTTGTCCAGTCTCC
	Trbv13-1+2	CCAGGGTTTTCCCAGTCACGACGAGGCTGCAGTCACCCAAAGCCC
	Trbv13-3	CCAGGGTTTTCCCAGTCACGACGAGGCTGCAGTCACCCAAAGTCC
	Trbv14	CCAGGGTTTTCCCAGTCACGACGAAGCTGGAGTCACCCAGTCTCC
	Trbv15	CCAGGGTTTTCCCAGTCACGACGATGCTGGAGTTACCCAGACACC
	Trbv16	CCAGGGTTTTCCCAGTCACGACAATGCTGGTGTCATCCAAACACC
	Trbv17	CCAGGGTTTTCCCAGTCACGACGATACTACGGTTAAGCAGAACCC
	Trbv19	CCAGGGTTTTCCCAGTCACGACGGTGGCATCATTACTCAGACACC
	Trbv20	CCAGGGTTTTCCCAGTCACGACGGAGCACTCGTCTATCAATATCC
	Trbv21	CCAGGGTTTTCCCAGTCACGACGACTCTGGGGTTGTCCAGAATCC
	Trbv23	CCAGGGTTTTCCCAGTCACGACGATGCTGCAGTTACACAGAAGCC
	Trbv24	CCAGGGTTTTCCCAGTCACGACGTTGCTGGAGTAACCCAGACTCC
	Trbv26	CCAGGGTTTTCCCAGTCACGACAATTCAAAAGTCATTCAGACTCC
	Trbv29	CCAGGGTTTTCCCAGTCACGACGACATGAAAGTAACCCAGATGCC
	Trbv30	CCAGGGTTTTCCCAGTCACGACAGTGTCCTCCTCTACCAAAAGCC
	Trbv31	CCAGGGTTTTCCCAGTCACGACGCTCAGACTATCCATCAATGGCC
2nd PCR	Linker	CCAGGGTTTTCCCAGTCACGAC

Purple letters indicate the linker sequence.

Supporting Table S3: TCRα primers for cloning (Vα gene specific PCR)

Name	5´-3´ nucleotide sequence
**Reverse**	
TRAC-REVin	CTGTCCTGAGACCGAGGATC (same as reverse primer from 2nd PCR)
**Forward (For cloning of the following gene segments into TRAV specific vectors)**	
Trav-2	**TGCAGCCATACAAACATTGCTACAAGCGAG**GTACATCTACTGGTACCGACAGG
Trav21	**TGTAGC CACGCCACAATCAGTGGAAACGAG**GTACATCTATTGGTACCGACAGG
Trav5-1	**TCAACTGCTCCTACACAGACAGTGCCTCTG**TCTACTTCCCTTGGTATAAGCAAG
Trav5d-3	**CTGCACTTATGTAGACAGTGCCTCTCTCTG**CTTCCCTTGCTATAAGCAAG
Trav5d-4	**ATCAACTGCACTTATGAGAACAGTGCCTCG**AACTACTTCCCTTGGTATAAGCAAG
Trav6-1	**ATTTATA AACTGTACTTATTCAACCACATGG**TACCCGACTCTTTTCTGGTA
Trav6-2	**ATTTATA AACTGTACCTATTCAACCACAGGG**TACCCGACTCTTTTCTGGTA
Trav6-3	**ATTTATA AACTGCACTTATTCAACCACAGGG**TACCCGACTCTTTTCTGGTA
Trav6-4	**ATTTATAAATTGCACTTATTCTACCACAACG**TACCCAACTCTTTTGTGGTA
Trav6-5	**GACTGTG AACTGTTCCTATGAAACCAAACAG**TACCCAACCCTGTTCTGGTA
Trav6-6	**TAAATTGCACGTATTCAGCCACAAGCATAG**CTTACCCTAATCTTTTCTGGTA
Trav6-7	**ACGATACACTGCAACTACTCAGCCTCA GGG**TACCCAACCCTGTTCTGGTA
**Forward (For cloning of the following gene segments into TRAV universal vector)**	
Trav1	**CTAGGCCTTCACCTAGCTGGGGTGAATGGG**CAGGGTGTGGAGCAGCCTGCCAA
Trav3	**CTAGGCCTTCACCTAGCTGGGGTGAATGGG**GAGCAGGTGGAGCAGCGCC
Trav4-2	**CTAGGCCTTCACCTAGCTGGGGTGAATGGG**ATGCCGGTGGAACAGAATCCTCCAGCCCTGAGTCTTTATGAAGGAGCTGAGTCTGGT CTGAGATGCAATT
Trav4-3	**CTAGGCCTTCACCTAGCTGGGGTGAATGGG**GATAAGGTGAAACAAAGTCCCTCAGCGCTGAGTCTCCAAGAAGGAACCAATTCTGCT CTGAGATGCAATT
Trav4-4	**CTAGGCCTTCACCTAGCTGGGGTGAATGGG**GATCAGGTGGAGCAGAGTCCTTCAGCCCTGAGCCTCCACGAGGGAACCGGTTCTGCTCTGAGATGCAATT
Trav7-2/3/4/5	**CTAGGCCTTCACCTAGCTGGGGTGAATGGG**CAGCAGAAGGTGCAGCAGAGCCCAGAATC
Trav7-6	**CTAGGCCTTCACCTAGCTGGGGTGAATGGG**CAGGAGAAGGTACAGCAGAGCCCAGAATC
Trav8-1	**CTAGGCCTTCACCTAGCTGGGGTGAATGGG**CAACTAGCAGAAGAGAATTCGTGGGCCCTGAGCGTCCACGAGGGTG
Trav8-2	**CTAGGCCTTCACCTAGCTGGGGTGAATGGG**CAATGGGGAGAAGAGAATCTTCAGGCTCTGAGCATCCAGGAGGGTG

Trav9-1	CTAGGCCTTCACCTAGCTGGGGTGAATGGGCAGACAGTTTCCCAGTCTGATGCCCATGTCACTGTCTTCGAAGGAGACTCGGTGGAGCTGAGATGCAACTATTCC
Trav9-2/3/4	CTAGGCCTTCACCTAGCTGGGGTGAATGGGCAGTCAGTGACACAGCCCGATGCTCGCGTCACTGTCTCTGAAGGAGCCTCTCTGCAGCTGAGATGCAAGTATTCC
Trav10	CTAGGCCTTCACCTAGCTGGGGTGAATGGGGAGAAGGTCGAGCAACACG
Trav11	CTAGGCCTTCACCTAGCTGGGGTGAATGGGAAGACCCAAGTGGAGCAGAGTC
Trav12-1/2+Trav12d/n-3	CTAGGCCTTCACCTAGCTGGGGTGAATGGGGACTCCGTGACCCAGACAGAAGGCCTGG
Trav12-3-001	CTAGGCCTTCACCTAGCTGGGGTGAATGGGGACTCAGTGACCCAGAAGGAAGGCCTGG
Trav13d-1	CTAGGCCTTCACCTAGCTGGGGTGAATGGGCAGCAAGTGCAGCAGAGTCCCACGTCCTTGGTTCTGCAGGAGG
Trav13d/n-3	CTAGGCCTTCACCTAGCTGGGGTGAATGGGCAGCAAGTGCAGCAGAGCCCCCCCGCGTCCTTGGTTCTGCAGGAGG
Trav13-1-201,13-2,13d/n2,13-4,13d/n4	CTAGGCCTTCACCTAGCTGGGGTGAATGGGCAGCAAGTGCAGCAGAGCCCCGCGTCCTTGGTTCTGCAGGAGG
Trav14d-1,14d/n-2,14-2-201,14d/n-3,14-3	CTAGGCCTTCACCTAGCTGGGGTGAATGGGCAGCAGCAGGAGAAACGTGACCAGCAGCAGGTGAGACAAAG
Trav14-1-201	CTAGGCCTTCACCTAGCTGGGGTGAATGGGCAGCAGAAGGAGAAACATGACCAGCAGCAGGTGAGACAAAG
Trav14-2-001	CTAGGCCTTCACCTAGCTGGGGTGAATGGGCAGCAGCAGGAGAAACATGACCAGCAGCAGGTGAGACAAAG
Trav15n/d-2-1	CTAGGCCTTCACCTAGCTGGGGTGAATGGGCAGAGAGTGACTCAGGTCCAGCCAACAGGCAGCAGTCAGTGGGGAGAAGAAGTCACC CTG GACTGTTCATATGAGACAAG
Trav15n-1	CTAGGCCTTCACCTAGCTGGGGTGAATGGGGAGAAAGTGATTCAGGTCTGGTCAACAGCAAGCAGGCAGGAGGGCGAAGAACTCACCCTGGACTGTTCATATGAGACAAG
Trav15-1-dv6-1-201	CTAGGCCTTCACCTAGCTGGGGTGAATGGGCAGAAAGTGATTCAGGTCTGGTCAACAACAAGCAGGCAGGAGGGCGAAAAACTCACACTG GACTGTTCATATAAGACAAG
Trav15-2-dv6-2-001	CTAGGCCTTCACCTAGCTGGGGTGAATGGGCAGAGAGTGACTCAGGTCCAGTCAACAGGCAGCAGTCAGTGGGGAGAAGTCACCCTGGACTGTTCATATGAGACAAG
Trav16	CTAGGCCTTCACCTAGCTGGGGTGAATGGGCAGAAGGTAACACAGACTCAGAC
Trav17	CTAGGCCTTCACCTAGCTGGGGTGAATGGGCAGTCCGTGGACCAGCCTGATGC
Trav18	CTAGGCCTTCACCTAGCTGGGGTGAATGGGCAACGGCTGGAGCAGAGTCCTCGGTTTCTGAG

Trav19	**CTAGGCCTTCACCTAGCTGGGGTGAATGGG**CAACAGAAGACTAGTGGCCAGCAAGTTAAACAAAGCTCTCC

Bold blue letters indicate the overlapping nucleotides for Gibson assembly cloning to the last nucleotides of the 5’ part of Vα gene segments in Trav specific vectors or the overlapping nucleotides for Gibson assembly cloning to the last nucleotides of the Trav14d-1 signal sequence in the Universal vector.

Bold yellow letters indicate the 5’ G nucleotide of the BamHI restriction site in the vector.

Red letters indicate the nucleotides overlapping with the 1st PCR primer-binding site.

Black letters indicate the nucleotides between the 1st PCR primer-binding site and the overlap with the corresponding vector sequence.

Supporting Table S4: TCRβ primers for cloning (Vβ specific PCR)

Name	5´-3´ nucleotide sequence
**Reverse**	
TRBC-REVin	CAAACAAGGAGACCTTGGGTGG (same as reverse primer from 2nd PCR)
**Forward**	
Trbv01SS	**CTCCTGTGTTCAAAACACATGGAGGCT**GACCCCACAGTGACTTTGCTGGAGCAAAACCC
Trbv02SS	**CTCCTGTGTTCAAAACACATGGAGGCT**AAAATTATCCAGAAACCAAAATATC
Trbv03SS	**CTCCTGTGTTCAAAACACATGGAGGCT**GGACCCAAAGTCTTACAGATCCC
Trbv04SS	**CTCCTGTGTTCAAAACACATGGAGGCT**GCTGTTTTCCAGACTCCAA
Trbv05SS	**CTCCTGTGTTCAAAACACATGGAGGCT**AACACTAAAATTACTCAGAAGATATCTAATCC
Trbv12-1SS	**CTCCTGTGTTCAAAACACATGGAGGCT**GATTCTGGGGTTGTCCAGTCTCC
Trbv12-2SS	**CTCCTGTGTTCAAAACACATGGAGGCT**AATTCTGGGGTTGTCCAGTCTCC
Trbv13-1+2 SS	**CTCCTGTGTTCAAAACACATGGAGGCT**GCAGTCACCCAAAGCCC
Trbv13-3SS	**CTCCTGTGTTCAAAACACATGGAGGCT**GCAGTCACCCAAAGTCCAAG
Trbv14SS	**CTCCTGTGTTCAAAACACATGGAGGCT**GGAGTCACCCAGTCTCCTCTCC
Trbv15SS	**CTCCTGTGTTCAAAACACATGGAGGCT**GGAGTTACCCAGACACCCAG
Trbv16SS	**CTCCTGTGTTCAAAACACATGGAGGCT**GGTGTCATCCAAACACCTAG
Trbv17SS	**CTCCTGTGTTCAAAACACATGGAGGCT**ACGGTTAAGCAGAACCCAAG
Trbv19SS	**CTCCTGTGTTCAAAACACATGGAGGCT**GATGGTGGCATCATTACTCAGACACC
Trbv20SS	**CTCCTGTGTTCAAAACACATGGAGGCT**GCACTCGTCTATCAATATCCCAG
Trbv21SS	**CTCCTGTGTTCAAAACACATGGAGGCT**GACTCTGGGGTTGTCCAGAATCC
Trbv23SS	**CTCCTGTGTTCAAAACACATGGAGGCT**GCAGTTACACAGAAGCCAAG
Trbv24SS	**CTCCTGTGTTCAAAACACATGGAGGCT**TAACCCAGACTCCACGATACC
Trbv26SS	**CTCCTGTGTTCAAAACACATGGAGGCT**AAAGTCATTCAGACTCCAAGATATC
Trbv29SS	**CTCCTGTGTTCAAAACACATGGAGGCT**AAAGTAACCCAGATGCCAAG
Trbv30SS	**CTCCTGTGTTCAAAACACATGGAGGCT**GTCCTCCTCTACCAAAAGCC
Trbv31SS	**CTCCTGTGTTCAAAACACATGGAGGCT**CAGACTATCCATCAATGGCC

Bold blue letters indicate the overlapping nucleotides for Gibson assembly cloning to the last nucleotides of Trbv13-2 signal sequence in the 2A-Linker.

Red letters indicate the nucleotides overlapping with the 1st PCR primer-binding site.

Black letters indicate the nucleotides between the 1st PCR primer-binding site and the overlap with the Universal vector sequence or to extended primer sequences for similar annealing temperatures.

Supporting Table S5: pMSCV based vectors for TCR expression cloning

Name of the vector	Used for insertion of a TCR with the following Vα- segment
Universal vector	Trav1, Trav3, Trav4-2, Trav4-3, Trav4-4, Trav7-2/3/4/5, Trav7-6, Trav8-1, Trav8-2, Trav9-1, Trav9-2/3/4, Trav10, Trav11, Trav12-1/2+Trav12d/n-3, Trav12-3-001, Trav13d-1, Trav13d/n-3, Trav13-1-201,13-2,13d/n2,13-4, 13d/n4, Trav14d-1,14d/n-2,14-2-201,14d/n-3,14-3, Trav14-1-201, Trav14-2-001, Trav15n/d-2-1, Trav15n-1, Trav15-1-dv6-1-201, Trav15-2-dv6-2-001, Trav16, Trav17, Trav18, Trav19
Trav-2 specific vector	Trav-2
Trav21 specific vector	Trav21
Trav5-1 specific vector	Trav5-1
Trav5d-3 specific vector	Trav5d-3
Trav5d-4 specific vector	Trav5d-4
Trav6-1 specific vector	Trav6-1
Trav6-2 specific vector	Trav6-2
Trav6-3 specific vector	Trav6-3
Trav6-4 specific vector	Trav6-4
Trav6-5 specific vector	Trav6-5
Trav6-6 specific vector	Trav6-6
Trav6-7 specific vector	Trav6-7

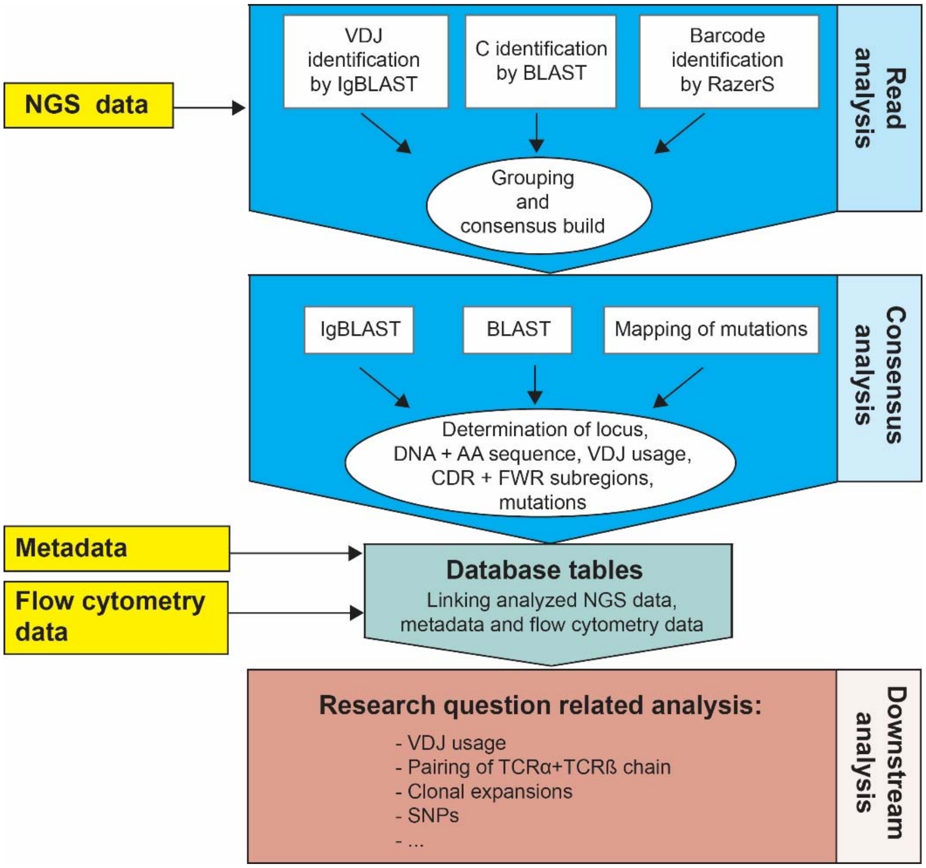

Supporting Fig. S1: Schematic view of the sciReptor T cell repertoire analysis pipeline

NGS data are analyzed at single-read level. Consensus sequences for reads from the same well, as annotated based on the unique barcode combinations, and locus are build and analyzed. A relational database connects the sequence information and the flow cytometric data for each cell with the sample metadata enabling downstream analyses.

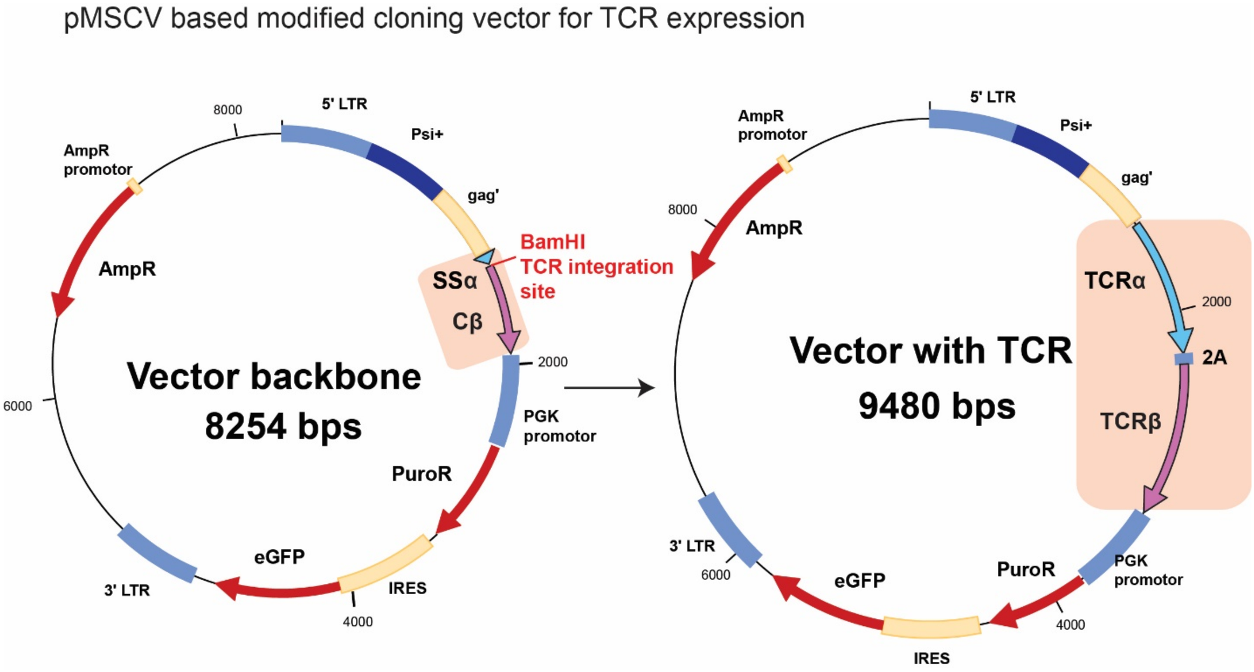

Supporting Fig. S2: Schematic view of the expression vector before and after head-to-tail insertion of the TCRα and TCRβ genes fused by the 2A linker

The pMSCV-based vector backbone contains a unique BamHI restriction site downstream of SSα and upstream of the Cβ encoding region for linearization and Gibson assembly with the TCRα, TCRβ and 2A-linker fragments. It also contains an independent eGFP-expression cassette that enables the identification of successfully transduced cells by flow cytometry and a puromycin-resistence gene for the selection of transduced cells independently of their TCR expression.

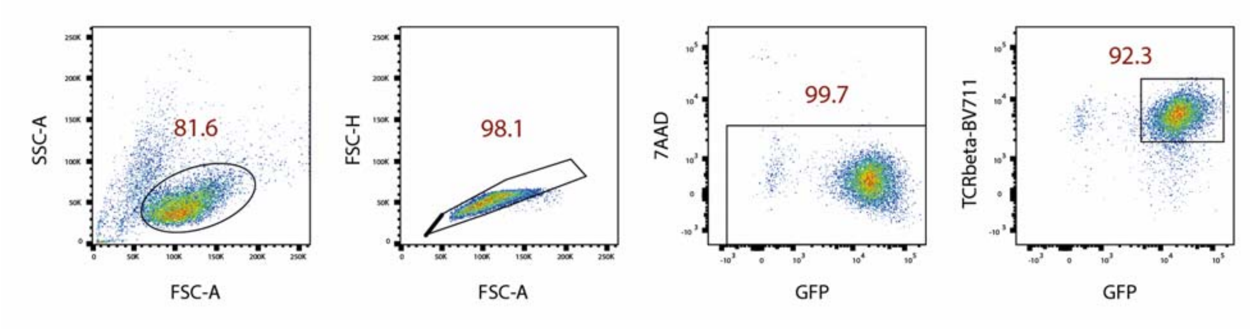

Supporting Fig. S3: Gating strategy for the analysis of TCR surface expression

Representative full flow cytometric gating strategy for the analysis of TCR surface expression levels of retrovirally transduced T cells. T cells are gated according to Forward and Sideward Scatter (FSC-A and SSC-A), duplicates are excluded by size parameters (FSC-H and FSC-A), live cells are gating by 7AAD^{+} cell exclusion, and T cells which are transduced are gated by GFP and TCRβ.

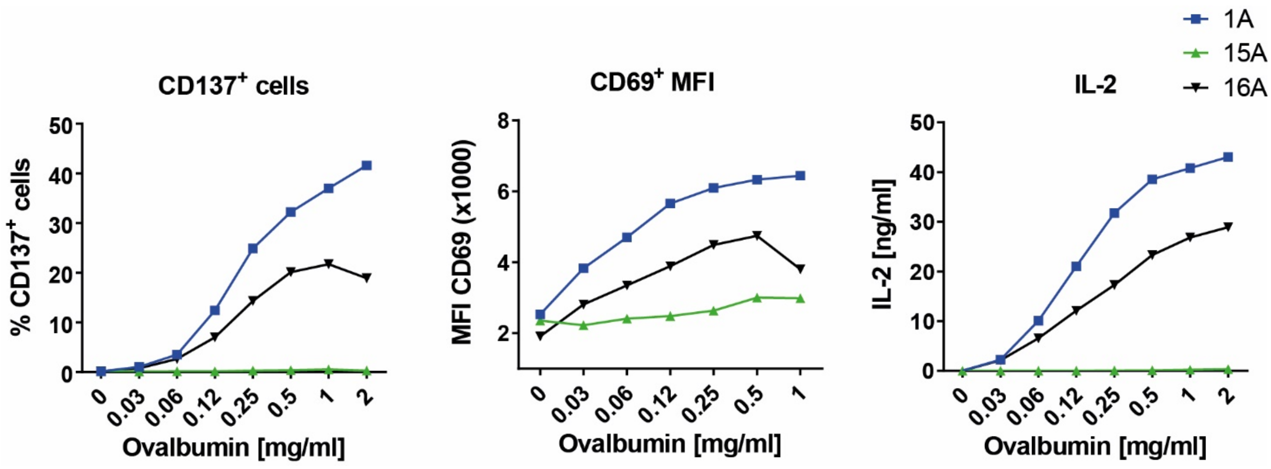

Supporting Fig. S4: TCR reactivity assay measured by different readout methods

Qualitative differences in the activation of CD4^{+} 58αβ$^{-/-}$ T cell lines expressing OVA-reactive TCRs cloned form primary T cells of OVA-immunized mice after stimulation with OVA-pulsed 771 B cells were measured based on the frequency of CD137^{+} cells and mean fluorescence intensity (MFI) of CD69 surface expression level in flow cytometry or by IL-2 concentration in culture supernatants using ELISA. Data are representative for one out of three independent experiments.
